# Biomaterial-Related Infections

**DOI:** 10.3390/jcm9030722

**Published:** 2020-03-07

**Authors:** Natália Martins, Célia F. Rodrigues

**Affiliations:** 1 Faculty of Medicine, University of Porto, Alameda Prof. Hernâni Monteiro, 4200-319 Porto, Portugal; 2 Institute for Research and Innovation in Health (i3S), University of Porto, 4200-135 Porto, Portugal; 3 LEPABE—Laboratory for Process Engineering, Environment, Biotechnology and Energy, Faculty of Engineering, University of Porto, Rua Dr. Roberto Frias, 4200-465 Porto, Portugal

Medical devices are a typical and important part of health care for both diagnostic and therapeutic purposes. Nonetheless, these devices (e.g., catheters, implants, dentures, or prostheses) recurrently lead to the appearance of several types of infections. In fact, there is a high rate of colonization of abiotic surfaces (such as biomaterials from medical devices), due to an induction of biofilm-growing microorganisms, which are progressively resistant to antimicrobial therapies. The biofilm structures are composed of attached and structured microbial communities, surrounded by an exopolymeric matrix. They are the predominant mode of microbial growth, as they offer ecological advantages, such as protection from the environment, nutrient availability, metabolic cooperation, and acquisition of new traits. Furthermore, there are single and multiple-species communities of biofilms, most of them particularly difficult to eradicate and a source of many recalcitrant infections. Undeniably, it is now recognized that most infections are connected to a biofilm etiology. 

Numerous methods have been established to fight device-related infections. Among them, there are natural products (e.g., phenolic compounds), surface coating/functionalization of biomaterials (e.g., peptides, β-lactams), or inorganic elements (e.g., copper and silver nanoparticles). These options are recognized mainly as having a broad-spectrum bacterial/fungal activity, being decisive to understand how these infections develop and to progress/find new biomaterials. Antifouling coatings (e.g., repellents or low adhesion to microorganisms, or antimicrobial coatings), improvement of biomaterials’ functionalization strategies, and support tissues’ bio-integration are some of them.

Eight papers were published in this issue, six of them being research papers with promising new developments. The reports describe the bioactivity of amorphous titania nanoporous and nanotubular coatings [1], the use of a method to increase the antimicrobial efficiency of a cold atmospheric plasma jet (CAPJ) [2], an electrospinning technique to acquire anti-infective terephthalate nanofibers loaded with silver nanoparticles [3], or the use of similar silver nanoparticles on the surface of titanium alloy implants, discussing nanotechnology and the antimicrobial effect of biomaterials [4]. Another report evaluated the effect of autoclaving sterilization in several parameters (such as morphology or biocompatibility) of implants modified by nanocomposite coatings [5], and, finally, a report focused on the efficacy of echinocandins (first-line antifungal drugs) for the treatment of systemic fungal infections derived exclusively from biofilm cells (mimicking a catheter-derived biofilm infection). Regarding reviews, two papers were published. The first one discussed the occurrence of candidiasis infections in diabetes mellitus (DM) and its complications (such as species, hospitalization, organs involved), and the second one discussed the management of *Streptococcus mutans–Candida* spp. oral biofilms’ infections, and the latest chemical and natural drugs used for this. These papers, which address the medical implications of the topics covered, will be summarized in the following lines. 

Piszczek et al. [1] concluded that surface-modified titanium alloy implants present the most suitable physicochemical and biological properties for a potential orthopedic application, with the important advantage of not having long-term release of mutagenic substances. Other work explains that CAPJ can destroy the *Escherichia coli* cell wall and damage its DNA structure, offering effective antimicrobial activity and being a new and significant approach to fight bacterial infections [2]. Likewise, terephthalate nanofibers loaded with silver nanoparticles have been indicated as a possible new approach in anti-infective therapy against Gram-positive and Gram-negative bacteria and fungi for wound dressings or implant coatings. The silver-decorated fibers revealed low cytotoxicity and inflammatory effects and, importantly, increased antibiofilm activity, stressing the anticipation of the use of these systems with antimicrobial activity [3]. A method for assembling two different systems of dispersed silver nanoparticles [4] has proved useful against Gram-positive and Gram-negative bacteria and yeasts. The results indicate high biocidal properties and biocompatibility (low toxicity) of the studied systems (particularly for one, Ti6Al4V/TNT5/0.6AgNPs). In another paper [5], the same authors describe the morphology, structure and mechanic alterations of nanotubular titania coatings, related to the autoclaving processes. They reveal that this sterilization method does not affect its morphology and structure, but it requires the elimination of adsorbed water particles from its surface, in order to avoid damage to the architecture of nanotubular coatings. The last research work is related to the efficacy of the treatment of an *in vivo* infection originated from *Candida glabrata* biofilm cells. Rodrigues et al. [6] indicated that caspofungin or micafungin does not have a significant impact on liver and kidney fungal burden or in the recruited inflammatory infiltrate (immune response). These results underline the greater virulence of biofilms cells’ infections (e.g., originating from medical devices), when compared to their planktonic counterparts.

Regarding reviews, both papers were related to fungal biofilms [7,8]. The first one assessed the incidence and prevalence of several *Candida* spp. infections in DM patients. The authors show that DM clearly predisposes individuals to fungal infections, specifically related to *Candida* spp., due to the patient’s general state of immunosuppression. In fact, patients have longer hospitalization periods, and candidiasis cases are commonly associated with the prolonged use of indwelling medical devices. These issues increase the disease-management-associated costs. Lastly, an article emphasized and discussed the use of new synthetic and natural drugs, besides other strategies, with promising results for both *S. mutans–Candida* spp. oral mixed biofilms treatment and control. These biofilms (among the most common in oral infections) have undergone several studies, including innovative drugs/therapeutic methods (e.g., photodynamic therapy, several naturally-occurring biomolecules, and chlorhexidine added to silver nanoparticles), revealing different, but promising, clinical approaches [8].
